# Retraction: Han, H., et al. Contact/Release Coordinated Antibacterial Cotton Fabrics Coated with N-Halamine and Cationic Antibacterial Agent for Durable Bacteria-Killing Application. *Int. J. Mol. Sci.* 2020, *21*, 6531

**DOI:** 10.3390/ijms21249489

**Published:** 2020-12-14

**Authors:** International Journal of Molecular Sciences Editorial Office

**Affiliations:** MDPI, St. Alban-Anlage 66, 4052 Basel, Switzerland; ijms@mdpi.com

We have been made aware that a number of figures in the paper cited above [1] contain manipulation. In Figure 6a, parts of the “Cotton-3” is the same as “Cotton-4”; in Figure 6b, parts of the “Raw Cotton” is the same as “Cotton-1”, and “Cotton-1” and “Cotton-4” are identical. In accordance with our ethics procedures, an investigation was conducted. The authors have not been able to provide a satisfactory explanation for these irregularities and the journal’s Editors-in-Chief no longer have confidence in the conclusions of the paper. Therefore, to ensure the addition of only high-quality scientific works to the field of scholarly publication, this paper [1] is retracted and shall be marked accordingly. The authors agree to this retraction. 

We apologize to our readership that this went undetected until now.
